# Self-instability of finite sized solid-liquid interfaces

**DOI:** 10.1038/srep18466

**Published:** 2015-12-21

**Authors:** L.K. Wu, B. Xu, Q.L. Li, W. Liu

**Affiliations:** 1 Key Laboratory of Advanced Materials of Education of China, Tsinghua University, Beijing, 100084, China; 2 School of Material Science and Engineering, Tsinghua University, Beijing, 100084, China

## Abstract

In solid-liquid systems, macroscopic solids lose their equilibrium and melt in a manner that results in overall movement of the solid-liquid interface. This phenomenon occurs when they are subjected to temperature gradients or external stress, for example. However, many experiments suggest that the melting of nano- and micro-sized metallic nuclei follows a different process not described by traditional melting theory. In this paper, we demonstrate through simulation that the melting of solid nuclei of these sizes occurs via random breaches at the interfaces. Moreover, this breaching process occurs at the exact solid-liquid equilibrium temperature and in the absence of any external disturbance, which suggests the name “*self-instability*” for this melting process. We attribute this spontaneous instability to the curvature of the samples; based on the relationship between the sample’s instability and its curvature, we propose a destabilizing model for small systems. This model fits well with experimental results and leads to new insights into the instability behavior of small-sized systems; these insights have broad implications for research topics ranging from dendrite self-fragmentation to nanoparticle instability.

Nano- and micro-sized solid and liquid metallic particles are widely used as components or structures in self-assembled functional devices^1^, catalysts^2^, and drug delivery systems^3^ and even as the endoskeletons of soft robots^4^. However, when decreased to the nano- or micro-scale, the instability of these material systems can lead to problems such as the fragmentation of dendrite nuclei^5^ and degradation of laser-processed silver nanowire^6^. Therefore, gaining understanding of these phenomena is of fundamental importance and general utility.

The general theory, Lindmann’s melting rule^7^ (LMR), treats the instability of the solid-liquid (SL) system as a result of external driving forces (e.g., temperature/chemical variation or stress gradients) that begins with the migration of point defects or dislocations^8^. Basically, LMR attributes the stability of a system to the competition between the interfacial energy and volume energy of the system. Indeed, the prominent role that interfacial energy plays at small scales can generate many exotic phenomena, including surface premelting^9^,^10^ and depression of the melting temperature of nanoparticles^11^,^12^, but it cannot describe all phenomena in small-sized systems.

In our work, nano- and micro-sized solid nuclei were observed to lose stability in the absence of any external driving force; termed “self-instability,” this phenomenon cannot be explained by LMR. Based on theoretical analysis and computation simulations, we hypothesised that the SL interfacial curvature and thermal fluctuations are relevant to self-instability. The interfacial curvature is important in determining the stability of the system thermodynamically, such as in the cases of surface melting of lead inclusions^13^ and metastability of the solid phase in pores^14^, in which the dynamics of the SL interface are not fully considered. Moreover, in the case of phase transitions of similarly scaled systems, such as the formation and coarsening of small clusters in the two-dimensional Ising model^15^ and grain-boundary migration in a two-dimensional colloidal crystal system^16^, the initial surface curvature plays an important role in determining the evolution of the system profile and causes the interface to be microscopically rough.

On the basis of this understanding, we conducted a computational study of the self-instability behaviour of a finite-size SL system of aluminium and investigated the importance of the curvature and thermal fluctuations in the dynamic evolution of the SL interface. We started from different-sized cylindrical nuclei and performed molecular dynamics (MD) simulations at different temperatures. In these simulations, the curvature contribution was investigated separately by observing the shrinkage and growth of the nuclei. Then, the influence of fluctuation was studied using both MD and the finite difference method (FDM); the stochastic nature of the self-instability process was also studied. Finally, the dynamics of the interface was explored, including the detailed shape of the breach and the rupture time of solid nuclei.

Traditionally, in an infinite SL system (i.e., a flat interface), melting is generally understood as the overall movement of the interface in the solid direction when the temperature of the system exceeds its melting point; in the opposite case (i.e., solidification). The dynamic behaviour of the interface can be described by the following expression^17^,^18^,^19^,^20^,^21^,^22^:


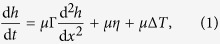


where *h*(*x*,*t*) is the profile of the interface, *x* is the distance along the interface, Δ*T* (=*T*–*T*_m_) is the undercooling and *η* is the thermal fluctuation. The SL interface exhibits atomically irregular roughness in Lennard-Jones^23^, hard sphere^24^,^25^, metal^26^,^27^ and alloy^28^,^29^ systems because of thermal fluctuations as illustrated in Fig. 1a. These fluctuations follow a Gaussian distribution and are included in the fluctuation term *η* (see Supplementary Theories and Supplementary Fig. S4 online). When an undercooling Δ*T* occurs, the interface velocity on the left-hand side is proportional to this thermodynamic driving force Δ*T* with a constant *μ*, which is referred to as the kinetic coefficient. Under equilibrium conditions (i.e., Δ*T* = 0), the profile of the interface is related primarily to the interfacial energy and to thermal fluctuations (see the first and second terms on the right-hand side of equation (1)). As a result, local interfacial concave and convex deviations will be introduced by the fluctuations; however, these temporary deviations will be straightened as much as possible by the interfacial tension, the effect of which is proportional to the interfacial energy, *Γ*, and to the second derivative of the interfaces, d^2^*h*/d*x*^2^. T*h*erefore, the interface will fluctuate near the equilibrium position indefinitely as shown in Fig. 1a. For convenience, we refer to the first and second terms in equation (1) as the interfacial energy term and the fluctuation term, respectively. In a finite SL system, however, the interface profile and dynamics are affected profoundly by the local curvature, and the dynamic evolution of the interface displays a different behavior. To illustrate these effects, the kinetic nature of the SL interface was amended, and a series of infinitely long, cylindrical Al solid nuclei inside the melt (see Fig. 1b) were simulated by MD (see methods). The results are described below.

## Results and Discussion

### Overall movement of the interface

In a finite SL system, the kinetic equation of the interface is written as


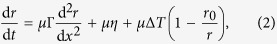


where instead of the interfacial profile being described as a function of *h*, it is described as a function of *r*, which is the radius of the sample. According to previous research^30^,^31^,^32^ and our results, the equilibrium temperature T* = *T*_m_–Δ*T* decreases with decreasing sample size *r*_0_ (see T*–*r* in the Supplementary Theories and Supplementary Table S1 online). Thus, each critical nucleus radius *r*_0_ exhibits a one-to-one correspondence with an undercooling Δ*T* condition. On the basis of the derivation shown in the Supplementary Theories online, we propose the third term in equation (2) to incorporate the curvature. Under the combined influence of the curvature and fluctuations, breaches can initiate on the SL interface at the equilibrium temperature and lead to the instability of the nuclei, which is discussed in a later section. Note that the interfacial profile *r* is treated as a function only of time *t* and distance along the centreline of the nucleus *x* in equation (2). In reality, it also depends on theta, the angle around the centreline, mainly because of the thermal fluctuations at the circumference. However, this factor does not significantly influence our results, and we relegate its discussion to the Supplementary Discussion online. Additionally, the extraction of the interfacial profile *r* is a critical process in simulations, the methods of which are provided in the Supplementary Methods online.

**Figure 2:**
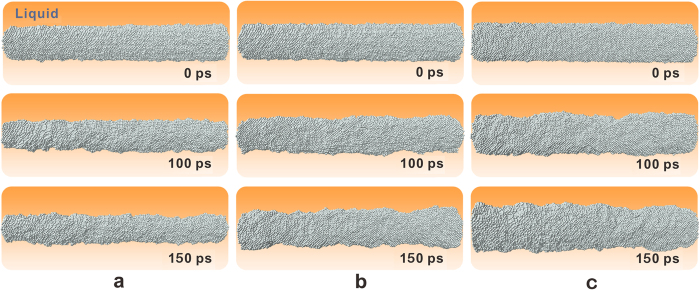
Snapshots of nuclei with different initial radius *r* at 892.975 K. **(a)** *r* = 28 Å; (**b**) *r* = 30 Å; (**c**) *r* = 32 Å. The silver balls represent solid atoms, and the orange sections represent the surrounding liquid phase.

**Figure 3:**
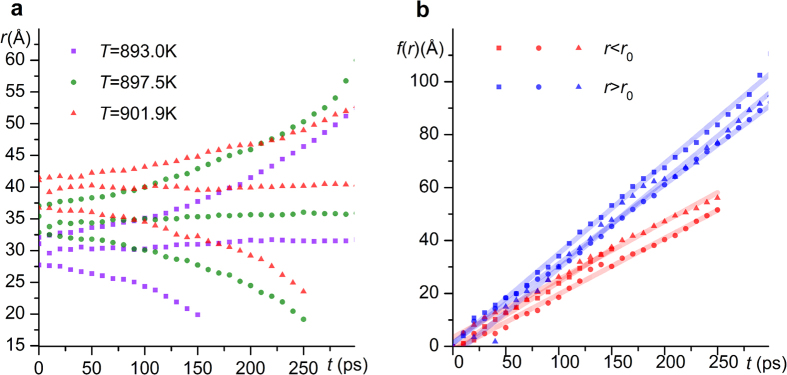
Radius of cylindrical nuclei (**a**) and the values of function *f*(*r*) (**b**) plotted vs. the relaxation time at various temperatures. The temperatures are indexed by the shape of the symbols in both figures. The functions of *f*(*r*) are plotted in red and blue for melting and solidification processes, respectively.

At temperatures that deviate from the equilibrium temperature (i.e., *r* ≠ *r*_0,T_), undercooling will induce an overall movement of the SL interface, similar to the flat interface. At three different temperatures T^*^ (892.975 K, 897.45 K, 901.9 K), MD simulations were performed for three samples with different critical sizes *r*_0,T_ (30 Å, 35 Å and 40 Å) with their centrelines lying along the [001] direction. When *r* = *r*_0,T_, the nuclei remained stable within our simulation timeframe. Furthermore, simulations were also carried out under T^*^ for samples with sizes smaller/larger than their corresponding *r*_0,T_; in these cases, the nuclei began to shrink (or grow). Fig. 2 shows the evolution of nuclei at 892.975 K. It was found that the nucleus with *r* = 30 Å (see Fig. 2b) remained stable to 150 ps, indicating that 30 Å is the critical size corresponding to 892.975 K; in the same time, the nucleus with *r* < 30 Å (as shown in Fig. 2a) shrank to approximately 20 Å and the nucleus with *r* > 30 Å (Fig. 2c) grew to approximately 40 Å. The same T^*^ for both the *r* = *r*_0,T_ and *r* ≠ *r*_0,T_ cases indicates that the contributions from the interface energy and thermal fluctuation terms are the same. Therefore, the driving force for interfacial movement originated only from the curvature term defined in equation (2) and the kinetic expression can be simplified to


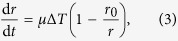


where positive and negative signs of velocity represent the solidification and melting processes, respectively. In the case of overall movement of the interface, the evolution of the radius with time can be described by solving the differential equation equation (3) and is expressed as


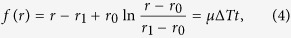


where *r*_1_ is the initial radius of the nucleus and *r*_0_ is *r*_0,T_. In these processes, the kinetic coefficient *μ* still dominates the intrinsic dynamic properties of interface movement and can be determined from the slope of the linear dependence of the function *f*(*r*) with respect to time *t*. The relationship between the radius of nuclei *r*, function *f*(*r*) and time *t* is shown in Fig. 3, from which different values of *μ* in the range of 892–902 K were calculated and summarized in Table 1; these values were observed to increase with increasing temperature. Moreover, the value of *μ* appeared to be larger in solidification than in melting, which requires further investigation.

### Stochastic instability of the critical nucleus

**Figure 4:**
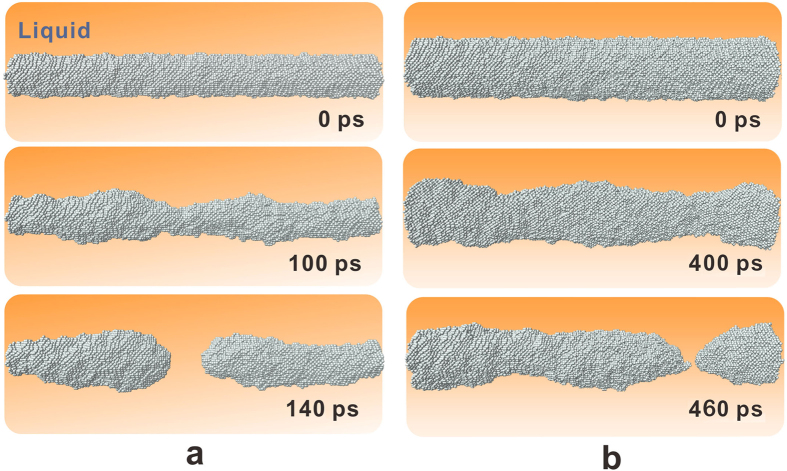
Snapshots of nuclei with *r* = 20 Å and 30 Å for different times at temperature *T* = T*. After the simulations were performed for a sufficiently long period, the nuclei became instable and broke into two parts.

At the equilibrium temperature T^*^, the nuclei with *r* = *r*_0,T_ should remain stable according to equation (2). However, the interface does not fluctuate ceaselessly as in the case of a flat interface. When we performed the simulation for a sufficiently long period, the nuclei lost stability. For example, although the nucleus of *r* = 20 Å exhibited an approximately cylindrical shape in the beginning of the simulation at 873.8 K, necks emerged on the interface shortly after 100 ps and formed breaches. The breaches grew continuously over time, and the nuclei eventually broke into two parts (see Fig. 4a). The same destabilization process occurred in all of the simulated nuclei, although more time was required for larger nuclei (140 ps for *r*_0,T_ = 20 Å and 460 ps for *r*_0,T_ = 30 Å). This finding implied that this instability behaviour was strongly dependent on the radius of the sample.

The size dependency of the splitting time originated from the competing effects of the interfacial energy and curvature terms in equation (2). These two terms play opposite roles in determining the interface profile. If a local deviation *δr* emerges on the interface, the interfacial energy term stretches it straight as much as possible, keeping the interface at its equilibrium position *r*_0,T_. However, the curvature term tends to drive the local interface farther away from the equilibrium position. In the beginning of the simulations, the fluctuation term led to the same amplitude *δr* and second derivative d^2^*r*/d*x*^2^ on the interfaces of a series of samples with various critical sizes; as a result, the same interfacial energy term and driving force tended to stretch the interface back to *r*_0,T_. For samples with smaller critical radii (i.e., a larger value of *δr*/*r*_0,T_ and a stronger curvature term), it was much easier for the local deviation to survive and grow, and rupture occurred more quickly. On the basis of this understanding, the process of instability originating from fluctuation can be summarized as follows. First, necks on the interface form randomly because of thermal fluctuations. Afterwards, these local profiles may be retained in the effects of the curvature term. As a result, a breach will start at a random position on the interface, extend gradually and lead to the rupture of the nuclei. This abrupt phenomenon can appear at any position of the interface because of the random characteristics of the fluctuation, such as in the middle (Fig. 4a) or one-quarter of the total distance from the end (Fig. 4b).

To clarify the effect of thermal fluctuations on interface instability, FDM simulations (see methods) were performed using four different routines. The radius of all nuclei was set at 100 Å, and the dynamics of these four experiments are described in Table 2. In simulations, the introduction of fluctuations was controlled, and perturbed interfaces were used to simulate the necked interface. When no thermal fluctuation was introduced, the sample with the straight interface maintained its initial profile, and no rupture occurred. If thermal fluctuations were added, the sample ruptured stochastically after 8.18 ns. The introduction of the perturbed interface (see Supplementary Methods online) can significantly reduce the rupture time, as shown in Table 2. The difference between cases 2 and 3 indicates that the time to form the perturbed interface represents more than half of the total rupture time. On the basis of these observations, the initial formation of the neck is an important step in the complete rupture process of the nuclei. This step constitutes most of the rupture time and determines the final location of the rupture. The details of these processes were shown in Supplementary Methods and Supplementary Fig. S9 online.

### Dynamics and interface morphology of the rupture

As discussed above, the rupture process could be separated into two steps: initial formation of a breach and extension of the breach. The fluctuation term and curvature term dominate these two processes, respectively. After the breach is formed, the effects of thermal fluctuations can be neglected, and the evolution of the interface is governed by interface and curvature terms as follows:


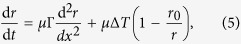


**Figure 5:**
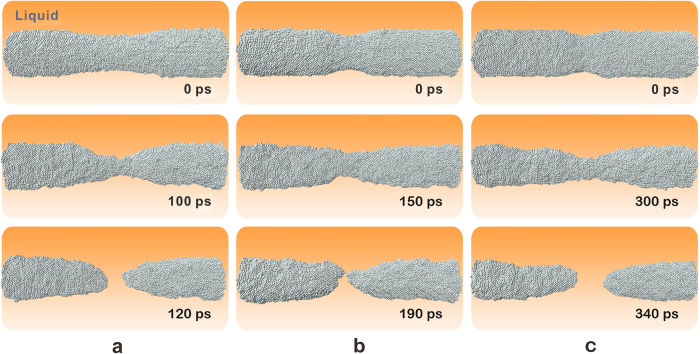
Snapshots of nuclei with *r* = 40 Å at 901.9 K. Breaches with the function *r* = 30 + *ax*^2^ were introduced artificially on the interface of nuclei. **(a)** *a* = 0.001; **(b)** *a* = 0.005; **(c**) *a* = 0.01.

Therefore, the subsequent rupture process is strongly dependent on the initial shape of the breaches. To obtain insight into the main microscopic mechanisms, we enhanced physical effects by simulating the extension process of breaches that were introduced artificially. The simulations were carried out by both MD and FDM calculations. The radii of the nuclei were *r* = 40 Å, and the corresponding equilibrium temperature was 901.9 K. The shape of the breaches was controlled by the function *r* = 30 + *ax*^2^, which means that all of the breaches had the same radius of *r* = 30 Å at the tip but different second derivative d^2^*r*/d*x*^2^ = *a*. As predicted by equation (5), smaller values of *a* induced faster nuclei splitting. Figure 5 shows snapshots of the breaching process of nuclei. For a smooth breach with *a* = 0.001, only 120 ps was required for nucleus rupture (Fig. 5a); as *a* was increased to 0.005 and 0.01, the rupture time increased to 190 ps and 340 ps, respectively.

**Figure 6:**
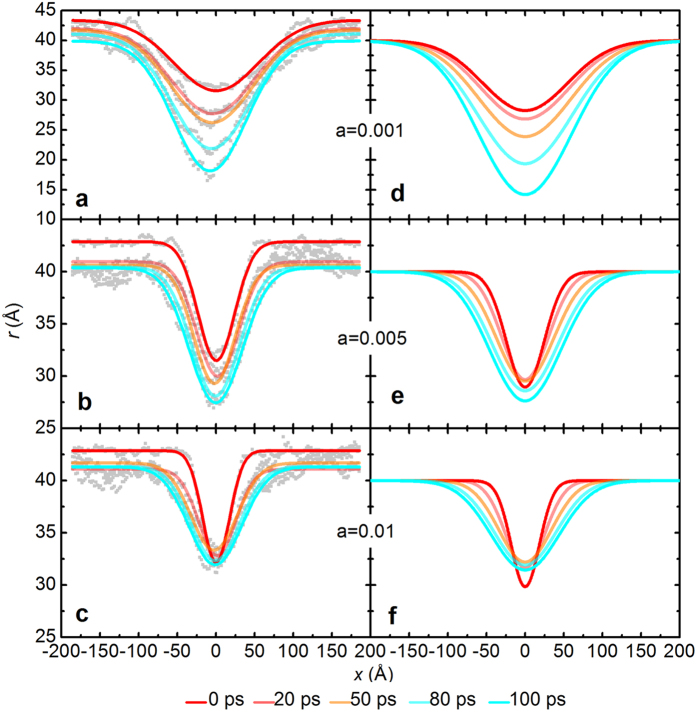
Interface evolution of the nuclei at 901.9 K. Breaches with the function *r* = 30 + *ax*^2^ (*a* = 0.001, 0.005, 0.01) were added to the interface. (**a–c**) MD simulations; (**d–f**) FDM simulations. Sampling times of 0, 20, 50, 80 and 100 ps were used. The appearance of the breaches follows an approximate Gaussian distribution.

To clarify the microscopic process of interface evolution, the interface profiles of the nuclei were extracted as shown in Fig. 6. For smooth breaches (i.e., smaller d^2^*r*/d*x*^2^), the tip of the breaches will move inward perpendicularly (as shown in Fig. 6a,d) because the stretching effect of the interfacial energy is too weak. For sharp breaches (i.e., larger d^2^*r*/d*x*^2^), the breach will first expand on both sides instead of moving forward, pulled by the interfacial tension. In this case, the value of d^2^*r*/d*x*^2^ decreases, and the breach will become shallow. Not until d^2^*r*/d*x*^2^ decreases to a certain value will the tip of the breach move inwards, as illustrated in Fig. 6c,f.

The fitting of the interface profile for a timeframe of 100 ps shows that it follows the shape of a Gaussian distribution. Indeed, the solutions of equation (5) (see Supplementary Methods online) provide an approximate Gaussian solution:


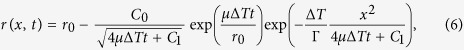


where *C*_0_ and *C*_1_ are constants that relate only to the initial interface morphology. The area and variance of the Gaussian distribution are proportional to time *t*, which can be considered as the inward movement and expansion of the breach, respectively.

### Rupture time

This self-instability process is difficult to observe because it occurs on a small scale; moreover, for smaller sizes, the entire process occurs more rapidly. One of the most favourable methods to observe melting phenomena involves synchrotron X-rays. The typical resolution of this method is on the order of several *μ*m. In our simulations, a series of studies was performed using FDM simulations to investigate the rupture time for nuclei from *r* = 10 Å to *r* = 10 *μ*m. All simulations started with initially perturbed interfaces. The results (see Supplementary Table S3 online) indicated that the rupture time for nuclei with *r* = 10 *μ*m was approximately 11.49 ms, which agrees well with experiment^33^ and is much faster than other previously proposed mechanisms^34^. From equation (6), an approximate relation between the rupture time and initial radius was obtained:


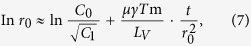


**Figure 7:**
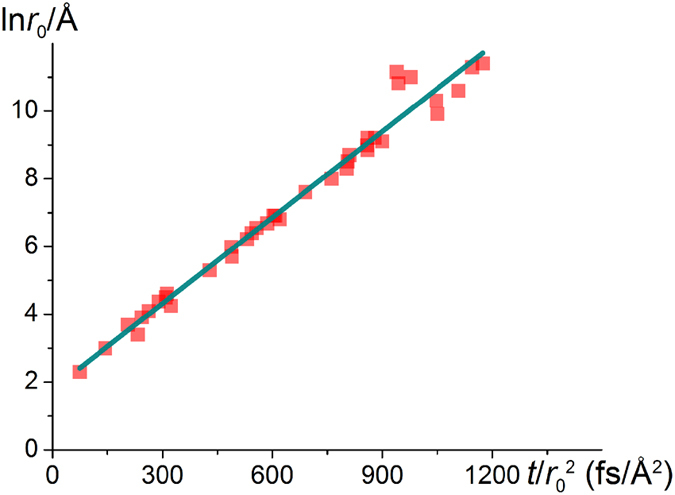
Relationship between ln*r*_0_ and *t*/*r*^2^_0_. A linear relationship is observed, and the parameters are in good agreement with the theoretical predictions.

On the basis of the material parameters we chose, the slope and intercept were estimated as 0.00915 Å^2^*/fs* and 1.42 *ln*(Å), respectively. (The derivation of equation (7) is presented in the Supplementary Discussion online). Figure 7 presents the fitting results for ln*r*_0_ and *t*/*r*_0_^2^, which can be expressed as


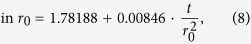


Thus, in industry, it is more practical to predict the rupture time by simply tuning the material parameters.

## Conclusion

In conclusion, we described a new type of melting phenomenon as a spontaneous self-instability of nano- and micro-sized metallic nuclei that requires no external driving force. The instability occurs locally and randomly on the interface of cylindrical nuclei with finite radius because of the interplay of the curvature and the thermal fluctuations. Based on this understanding, we constructed a model of nuclei melting dynamics, in which the curvature contribution was added to the existing LMR model. MD and FDM simulations confirmed this phenomenon and were in good agreement with the predictions of our model. Furthermore, the morphology of the interface and the melting time of this process were predicted; the results not only agree with the experimental results but also imply that the theory can be used widely in finite SL systems.

## Methods

### Molecular dynamics simulations

In our work, MD simulations were used to study the shrinkage, growth and instability of cylindrical nuclei. The simulations were carried out with LAMMPS. The potential in the simulations is Al1^35^, a type of EAM.fs potential from Mendelev that sets the melting point^35^ and the latent heat of fusion^36^ to be 926 K and 1011.55 mJ/mm^3^, respectively. NPT ensembles were employed, in which the Nose-Hoover thermostat and the Nose-Hoover barostat were used to control the temperature and pressure of the system, respectively. The simulation process (e.g., for *r* = 30 Å) was arranged as follows:

1. Initially, a system of 124.2 Å ×124.2 Å ×372.6 Å was selected, and 324,000 atoms were arranged in fcc lattices. The system was relaxed at 930 K over a period of 50 ps.
2. The atomic radius of *r* ≤ 30 Å was set for solid atoms at fixed position. Then, the atoms outside the solid nuclei were heated to 1150 K over a period of 300 ps; these atoms became the liquid phase as the temperature exceeded the melting point.
3. After a relaxation for 20 ps, the system was cooled to a temperature below the melting point over a period of 100 ps.
4. Afterwards, we monitored the movement of the SL interface. If the interface moved towards the liquid side, the nuclei grew, which indicates crystallization of the liquid phase; we then slightly increased the temperature, or vice versa. After a series of trial-and-error attempts, the interface became stationary at 892.975 K.
5. The radius of the nuclei was set to 28 Å, and the process was repeated from step (1) to step (4). The shrinkage of the nuclei was simulated; similarly, the growth of the nuclei was simulated by setting the radius to *r* = 32 Å.

### Finite difference method simulations

Because of the limits of MD simulations in size and duration, FDM simulations were performed according to equation (2). In the simulations, the nuclei were divided into a series of finite elements Δ*x,* and a timestep Δ*t* was used to update the local radii of the nuclei. The equation used in the simulations was obtained by amending equation (2) as


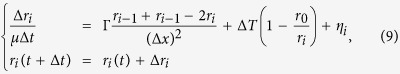


where *r*_0_ is related to Δ*T* (see supplementary theories). In this work, FDM simulations were used to study the self-instability of nuclei. The parameters used in the simulations, shown in Supplementary Table S2 online, were obtained from our previous work (unpublished).

## Additional Information

**How to cite this article**: Wu, L.K. *et al.* Self-instability of finite sized solid-liquid interfaces. *Sci. Rep.* **5**, 18466; doi: 10.1038/srep18466 (2015).

## Supplementary Material

Supplementary Information

## Figures and Tables

**Figure 1:**
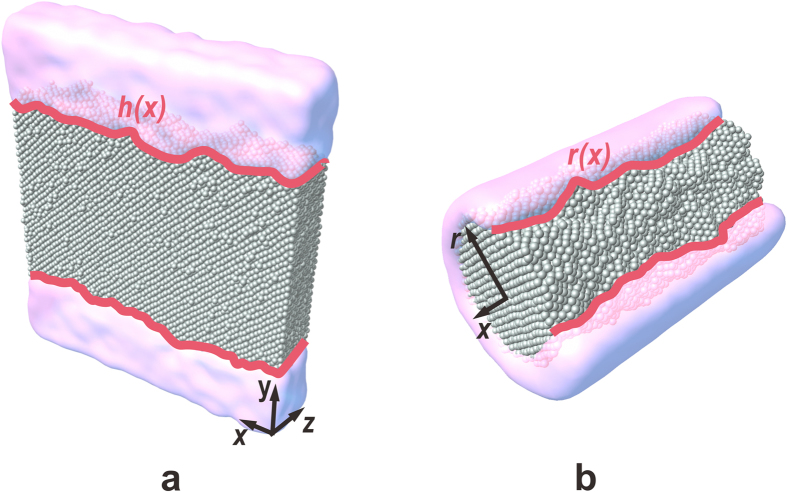
Schematic of interface fluctuations: (**a**) flat interface and (**b**) cylindrical interface. The silver balls represent solid atoms surrounded by a liquid phase coloured as pink.

**Table 1:** Kinetic coefficients calculated at various temperatures.

*T* (K)	*r*_1_ (Å)	State	*μ* (cm s^−1^ K^−1^)
892.975	28	shrinkage	75.57
	32	growth	90.78
897.45	33	shrinkage	76.71
	37	growth	119.22
901.9	38	shrinkage	87.85
	42	growth	137.96

The kinetic coefficient increases with increasing temperature in the range of 892 K to 902 K. At the same temperature, the coefficient from shrinkage is less than that from growth.

**Table 2:** Results of simulations of rupture of nucleus *r* = 100 Å.

Simulation	1	2	3	4
Rupture time	–	8.18 ns	3.12 ns	3.12 ns
Rupture position	—	random	fixed	fixed

Four types of simulations were performed. 1: Straight interface; 2: Straight interface + thermal fluctuation; 3: Perturbed interface; 4: Perturbed interface + thermal fluctuation.
