# Supplementary material for: Self-instability of finite sized solid-liquid interfaces
Source: Sci Rep. 2015 Dec 21;5:18466. doi: 10.1038/srep18466 (PMC4685266; doi:10.1038/srep18466)
Supplement: Supplementary Information [file srep18466-s1.pdf]

Supplementary Materials for

Self-instability of finite sized solid-liquid interfaces

L.K. Wu[1,2], B. Xu[1,2,*], Q.L. Li[1,2], W. Liu[1,2,*]

[1]Key laboratory of Advanced Materials of Education of China, Tsinghua University, Beijing, 100084, China

[2]School of Material Science and Engineering, Tsinghua University, Beijing, 100084, China

Ⅰ. Supplementary Figures and Tables,

Ⅱ. Supplementary Theories,

Ⅲ. Supplementary Methods,

Ⅳ. Supplementary Discussions,

Ⅴ. Supplementary References.

*Corresponding authors: xuben@mail.tsinghua.edu.cn; liuw@mail.tsinghua.edu.cn

Supplementary Figures and Tables

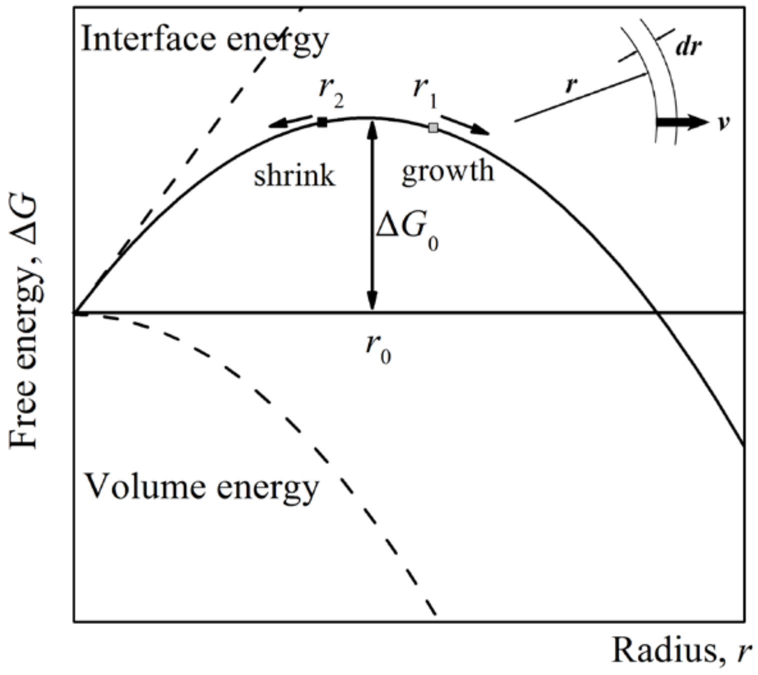

Supplementary Figure S1. The free energy Δ*G* is plotted as a function of the radius *r* of the cylindrical nucleus in the undercooled.

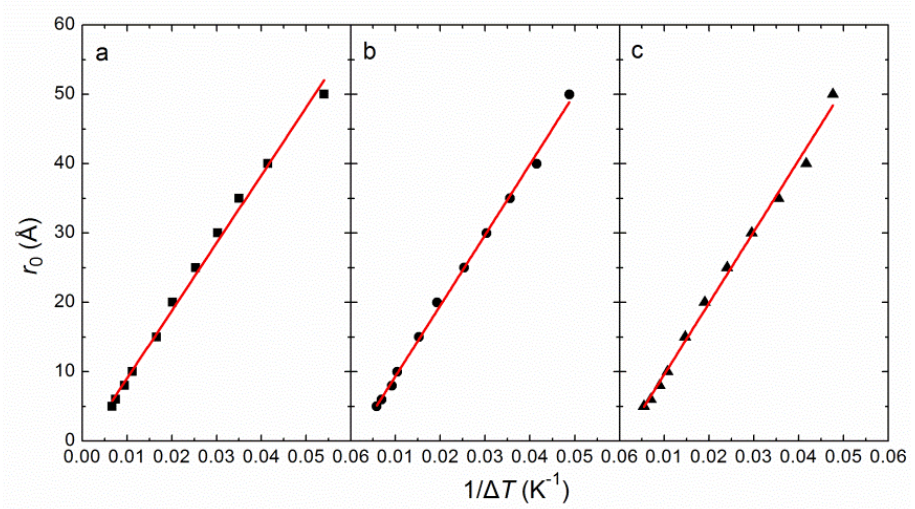

Supplementary Figure S2. The inverse relation between the critical nuclei size r_0 and the critical undercooling Δ*T*. Nuclei with their centrelines lying along the directions of [100] (a), [110] (b), [111] (c) were in equilibrium with liquid in MD simulations.

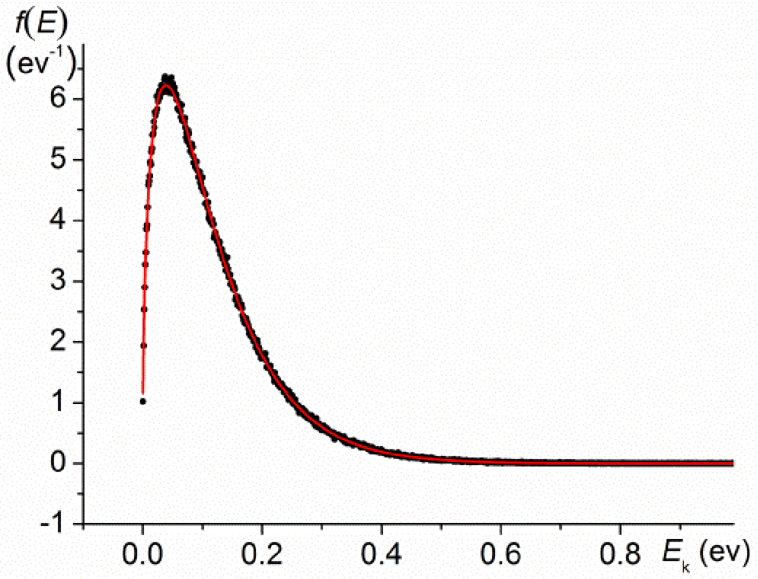

Supplementary Figure S3. The distribution of kinetic energy for surface atoms. 719371 interfacial atoms were counted. The black dots come from simulations, red line is the standard Maxwell distribution.

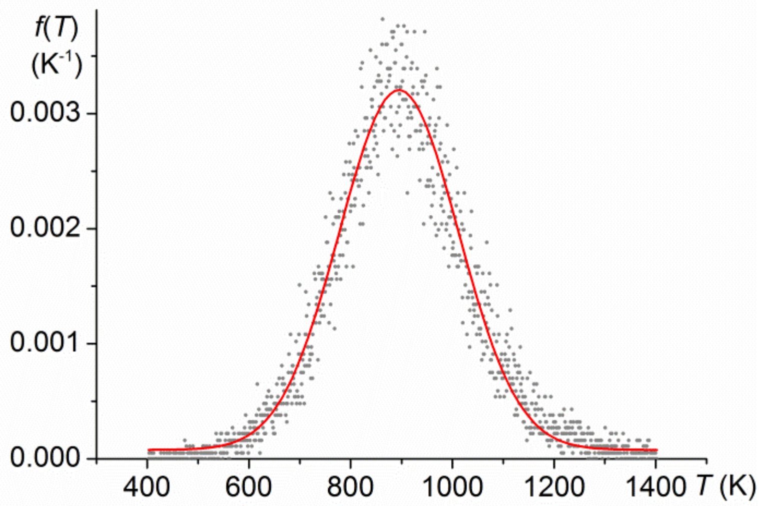

Supplementary Figure S4. The statistics of temperature distribution for finite elements of Δx=1 Å. 18600 interfacial finite elements were counted. The red line is the fitted line, follows the Gauss distribution.

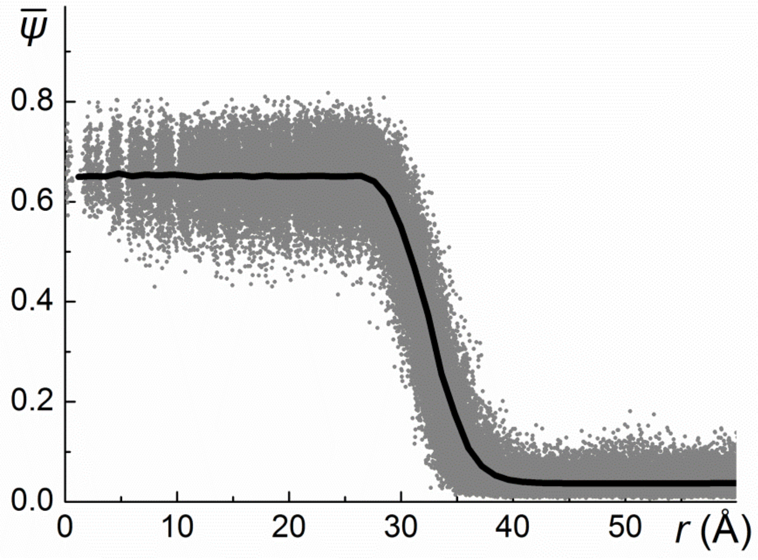

Supplementary Figure S5. The order parameter vs. radius for each atom in an instantaneous configuration of the nucleus *r* = 30 Å. The nucleus region, where the order parameter is larger, is solid phase, while the region where *r* > 30 Å, the parameter is small correspond to the liquid region.

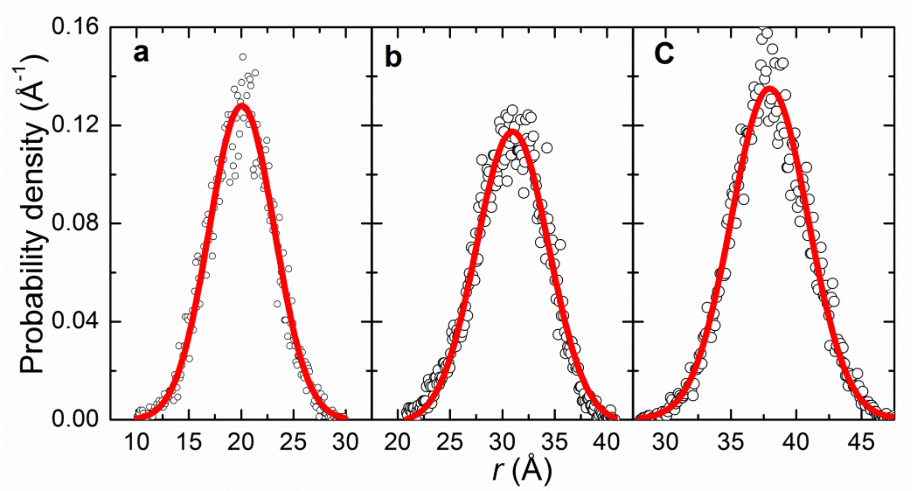

Supplementary Figure S6. The probability density distribution of interfacial atoms' radius. Three nuclei with **(a)** *r* = 28 Å, **(b)** *r* = 30 Å and **(c)** *r* =32 Å were relaxed at 892.975 K for 150ps. All of the probability density exhibit a good fit of Gauss distribution.

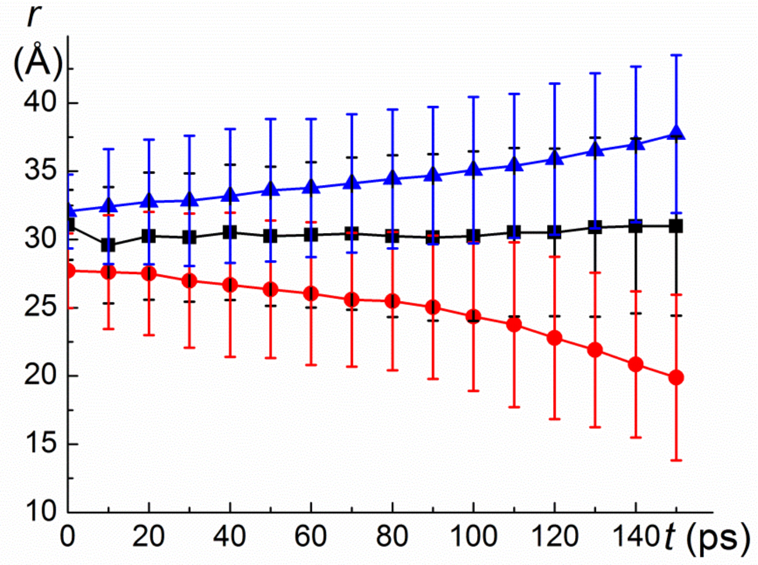

■ r = 30 Å; ● r = 28 Å; ▲ r = 32 Å.

Supplementary Figure S7. Radius of cylindrical nuclei vs. the relaxation time. Three nuclei r = 28 Å, 30 Å and 32 Å were relaxed at 892.975 K for 150 ps, and showed shrinkage, stable and growth, respectively. The error bars indicate the 95% confidence intervals.

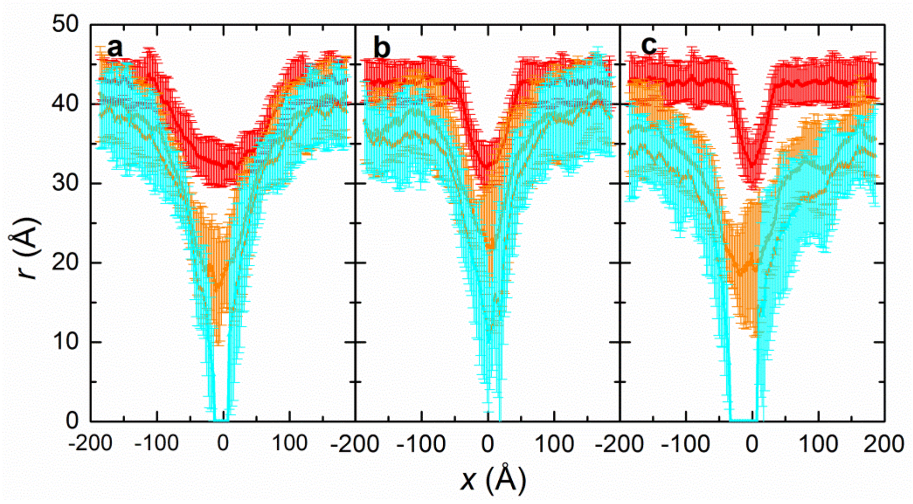

Supplementary Figure S8. The interfaces' evolution of the nuclei at 901.9K. Breaches as the function $r = 30 + ax^2$ (a = 0.001, 0.005, 0.01) were added to the interface. **(a)** a = 0.001, the time for sampling was 0 ps, 100 ps and 120 ps; **(b)** a = 0.005, the time for sampling was 0 ps, 150 ps and 190 ps; **(a)** c = 0.01, the time for sampling was 0 ps, 300 ps and 340 ps; The error bars

indicate the 95% confidence intervals.

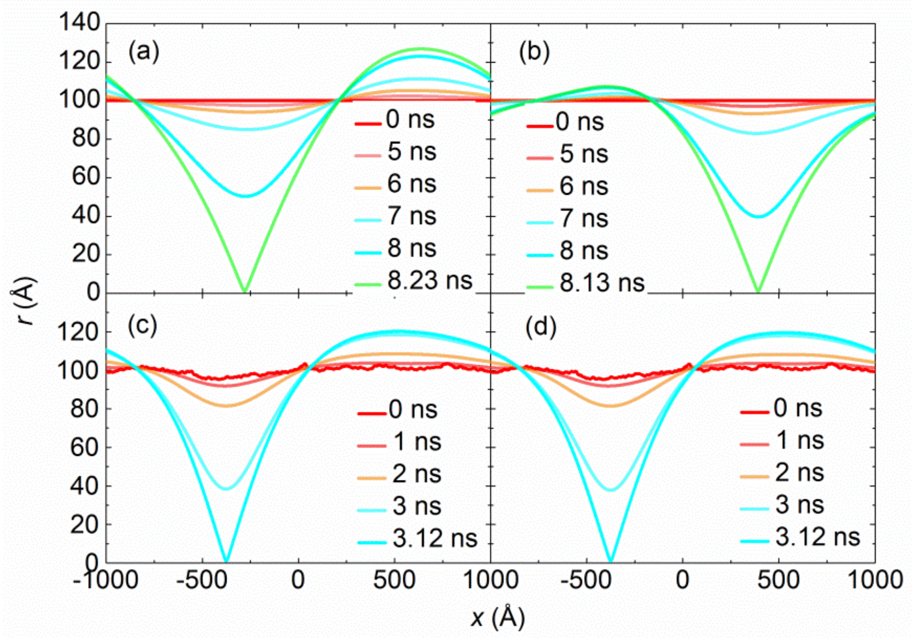

Supplementary Figure S9. The FDM simulations for nuclei *r*=100 Å. FDM simulations were performed under three sets of conditions. **a), b)** straight interface + thermal fluctuation, **c)** perturbed interface, and, **d)** perturbed interface + thermal fluctuation.

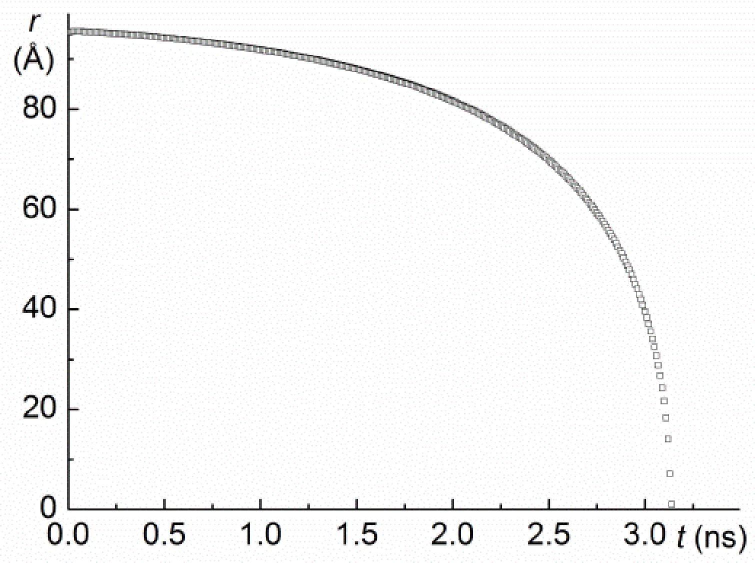

Supplementary Figure S10. The radius of the breach tip in FDM simulation. The initial radius of the nucleus is 100 Å. The nuclei ruptured in 3.12 ns, most time the radius was larger than 70 Å.

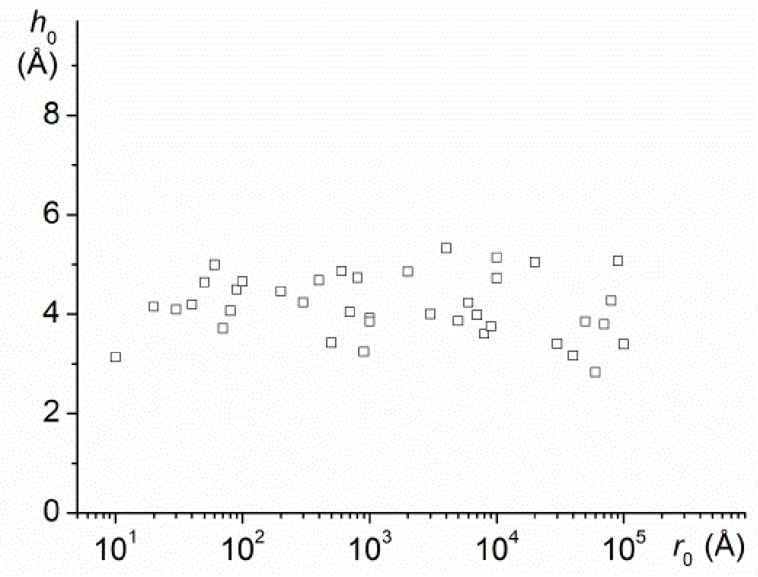

Supplementary Figure S11. The depths of the breach of nuclei from *r*=10 Å to *r*=10 μm. For the perturbed interfaces, the initial depths of the breach appeared near 4 Å, with an average of 4.15 Å.

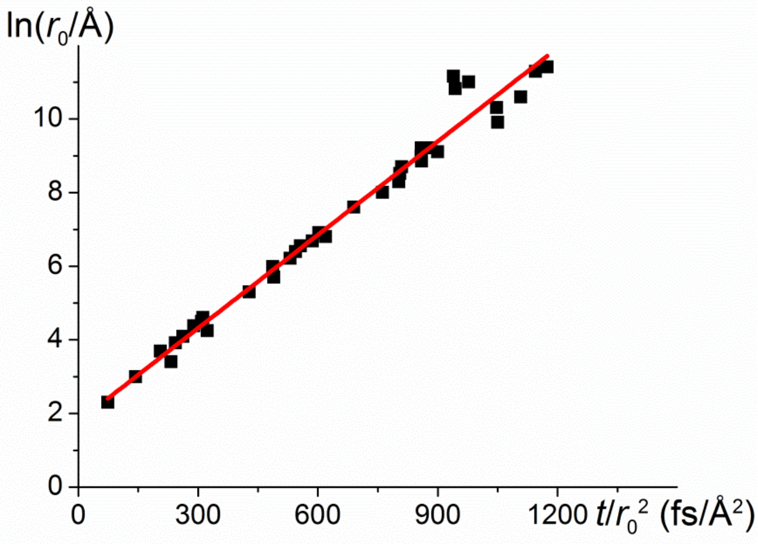

Supplementary Figure S12. Results by fitting lnr_0∝t/r_0^2. It fits well with a linear relationship, while the parameters are in good agreement with the theory predictions.

r_0 (Å)	$T_{[100]}$ (K)	$T_{[110]}$ (K)	$T_{[111]}$ (K)
5	776.78	755	744
6	793	782	784
8	820	817.5	815
10	836.8	830	833
15	865.95	860.5	858
20	876.42	874.1	873.455
25	886.6	886.5	884.4
30	892.975	893	892.1
35	897.45	897.85	897.8
40	901.9	901.9	902
50	907.5	905.5	905

Supplementary Table S1. The relations between the critical radius and the equilibrium temperatures. Three kinds of nuclei along the [100], [110], [111] directions were in equilibrium with their surrounding liquid in MD simulations.

T_m (K)	L_V (mJ/mm^3)	γ (mJ/m^2)	γ+γ'' (mJ/m^2)	Γ (Å•K)	μ (cm•s^{-1}•K^{-1})
**926**	1011.55	100	90	840	100

Supplementary Table S2. Calculation parameters used in the FDM. Γ was 843.29 Å K; a value of 840 was used for convenience. 100 cm s^{-1} K^{-1} was used for μ, between 75 and 140 in the MD simulations. See the interpretation of other parameters in the text.

r_0(Å)	10	20	30	40	50
t (fs)	7,417	57,544	209,245	329,317	609,045
r_0(Å)	60	70	80	90	100
t (fs)	945,294	1,583,744	1,851,608	2,501,170	3,123,106
r_0(Å)	200	300	400	500	600
t (fs)	17,133,182	44,078,519	77,975,370	132,708,048	195,926,880
r_0(Å)	700	800	900	1000	2000
t (fs)	272,814,404	375,214,641	501,851,297	602,863,329	2,758,629,100
r_0(Å)	3000	4000	5000	6000	7000
t (fs)	6,857,562,000	12,837,495,900	20,131,077,700	29,158,542,800	42,118,390,800
r_0(Å)	8000	9000	10000	20000	30000
t (fs)	54,918,320,200	72,885,888,300	87,946,829,200	420,118,670,000	943,095,040,000
r_0(Å)	40000	50000	60000	70000	80000
t (fs)	1,772,662,130,000	2,359,632,660,000	3,518,675,790,000	4,777,860,800,000	7,328,311,890,000
r_0(Å)	90000	100000			
t (fs)	9,508,772,310,000	11,486,606,050,000			

Supplementary Table S3. The time spent on the rupture of nuclei from *r*=10 Å to *r*=10 μm.

To make sure the length of nuclei long enough, they are selected as $20r_0$.

Supplementary Theories

The critical nuclei radius of cylindrical nuclei. According to the classical nucleation theory (CNT)[1~2], to form a small solid sphere of radius r in an undercooled liquid, the change of the Gibbs free energy can be expressed as:

$$\Delta G = -\frac{4}{3}\pi r^3 \Delta G_V + 4\pi r^2 \gamma, \quad (1)$$

where γ is the solid-liquid interfacial energy and ΔG_V is the Gibbs free energy difference per unit volume between solid and liquid phases at the same temperature. In CNT, the approximated ΔG_V can be expressed as:

$$\Delta G_V \cong L_V \frac{\Delta T}{T_m}, \quad (2)$$

where L_V is the latent heat of fusion per unit volume at the equilibrium meting point, T_m is the equilibrium melting point, and $\Delta T(=T_m\text{-}T)$ is the undercooling. The critical nucleus radius is obtained from equation (1):

$$r_0 = \frac{2\gamma}{\Delta G_V} \cong \left(\frac{2\gamma T_m}{L_V}\right)\frac{1}{\Delta T} \quad (3)$$

Considering an infinite long cylindrical nucleus, the difference in the Gibbs free energy and critical nuclei radius are amended as:

$$\Delta G = -\pi r^2 \Delta G_V + 2\pi r \gamma, \quad (4)$$

$$r_0 = \frac{\gamma}{\Delta G_V} \cong \left(\frac{\gamma T_m}{L_V}\right)\frac{1}{\Delta T} \quad (5)$$

For a given undercooling, there exists a critical radius r_0, as shown in Supplementary Fig. S1. If $r < r_0$, the nucleus shrinks in order to lower the free energy of the system; if $r > r_0$, it grows to lower the free energy; if $r = r_0$, the nucleus can be in equilibrium (but metastable) with its surrounding liquid.

In our work, the cylindrical nuclei of Al from r=5 Å to r=50 Å were calculated using MD

simulations. The potential in the simulations is Al1[3], a kind of EAM.fs potential from Mendelev, that sets the melting point[3] and the latent heat of fusion[4] to be 926 K and 1011.55 mJ/mm^3, respectively. Three kinds of nuclei with their centrelines lying along the [100], [110], [111] directions were in equilibrium with liquid. The results were shown in Supplementary Table S1, from which the interfacial energy can be obtained by equation (5). Supplementary Fig. S2 shows the relation between r_0 and $1/\Delta T$. The interfacial energies of the three nuclei were $\gamma_{[100]}$=106.74 mJ/m^2, $\gamma_{[110]}$=111.41 mJ/m^2, $\gamma_{[111]}$=112.72 mJ/m^2, calculated from the slope of the linear fitting.

The kinetic expression of interface fluctuation

A. Infinite flat interface. According to equation (5), ΔT is 0 for sample of infinite r_0 (flat interface), and the system keeps stable under this condition. Then if $T < T_m$, the interface will move toward the liquid phase with a velocity V[5~9]:

$$V = V_0 \left[1 - \exp(-\frac{Q}{k_B T}) \right], \tag{6}$$

where Q is the thermodynamic driving force, defined as the difference of free energy between solid and liquid phases per atom, V_0 is a temperature-dependent factor representing the maximum velocity, and k_B is the Boltzmann constant. For a flat interface, Q only includes the change of volume Gibbs free energy, which can be described as:

$$Q = \frac{\Delta G_V}{\rho} \cong \frac{L_V}{\rho} \frac{\Delta T}{T_m}, \tag{7}$$

where ρ is density of atoms.

In small undercooling limit, equation (6) can be linearized and yields:

$$V = \left[\frac{V_0 L_V}{\rho k_B T_m^2} \right] \Delta T = \mu \Delta T, \tag{8}$$

where the term multiplying the undercooling ΔT can be identified as the kinetic coefficient μ.

Under zero undercooling, the interface is stable, but the temperature fluctuation η introduces local movement of the interface with a velocity of $\mu\eta$. Therefore, the kinetic[10] of the interface can be expressed as:

$$\frac{\mathrm{d}h}{\mathrm{d}t} = \mu\Gamma\frac{\mathrm{d}^2h}{\mathrm{d}x^2} + \mu\eta, \quad (9)$$

where $h(x,t)$ is the profile of the interface, x is the direction along the interface, and Γ is related to the interfacial energy as follows:

$$\Gamma = \frac{\gamma + \gamma''}{L_V} T_m, \quad (10)$$

where $\gamma + \gamma''$ is called interfacial stiffness. As a result of equation (9), concave and convex will form at different positions on this overall stable interface.

B. Finite size effect on interface dynamics. In the finite case, free energy Q not only includes the volume free energy but also the interfacial energy. For instance, for the growth of radius from r to $r+\mathrm{d}r$, the difference in free energy is:

$$Q = (\Delta G_V - \frac{\gamma}{r}) \cdot \frac{1}{\rho}, \quad (11)$$

where r is the radius of an infinite long cylindrical nucleus. Therefore the velocity expression in equation (8) is amended as:

$$\frac{\mathrm{d}r}{\mathrm{d}t} = V = \mu\Delta T(1 - \frac{r_0}{r}) \quad (12)$$

Therefore, the nucleus $r = r_0$ is stable under certain ΔT. The interface will move as a whole once $r \neq r_0$ with a velocity V. As a consequence of equation (12), the kinetic expression of equation (9) should be amended as:

$$\frac{\mathrm{d}r}{\mathrm{d}t} = \mu\Gamma\frac{\mathrm{d}^2r}{\mathrm{d}x^2} + \mu\eta + \mu\Delta T(1 - \frac{r_0}{r}), \quad (13)$$

where interfacial profile is described by *r* instead of *h*, which is the radius of the nuclei.

What must be pointed out that the radius *r* in equation (13) is treated just as a function of time *t* and the distance along the centerline of the nucleus *x*. However, it is also depends on θ, the angle around the centerline of the nucleus, which will be discussed in the later section (see Supplementary Discussion).

Temperature profile of the interface atoms. According to statistical physics, the kinetic energy of atoms follows the Maxwell distribution:

$$f(E)=\frac{\mathrm{d}N_E}{N\mathrm{d}E}=\frac{2}{\sqrt{\pi}}(\frac{1}{k_B T})^{\frac{2}{3}}\exp(-\frac{E}{k_B T})E^{\frac{1}{2}}, \tag{14}$$

where the kinetic energy has relation to temperature by $E_i=3k_BT_i/2$, *T* is the temperature of atom *i*. In our work, MD simulations of nuclei were simulated to obtain the statistic data of kinetic energy of interfacial atoms, defined by Morris[11] (see Supplemental Methods). In Supplementary Fig. S3, 719371 interfacial atoms were counted. The black dots are statistics, while red line is the Maxwell distribution. There is good consistency between them. The mean and variance of Maxwell distribution are:

$$\begin{cases} \mu=\frac{3}{2}kT \\ \sigma^2=\frac{3}{2}(kT)^2 \end{cases} \tag{15}$$

If there are *n* atoms exist on a finite element, Δx, along the centerline of the nucleus. The mean kinetic energy of these atoms follows the Gauss distribution according to the central limit theorem:

$$\frac{\frac{3}{2}k_BT-\frac{1}{n}\sum_{i=1}^{n}E_x}{\sqrt{\frac{3}{2}}k_BT/\sqrt{n}} \propto N(0,1), \quad (16)$$

Therefore, the temperature of this element Δx should be:

$$\frac{T-T_x}{\sqrt{\frac{2}{3}}T/\sqrt{n}} \propto N(0,1) \quad (17)$$

where T-T_x is thermal fluctuation (η in equation (13)) at x, represents the temperature difference between the local atoms and the system. The number of interfacial atoms, n, can be obtained by:

$$n = 2\pi r_0 \Delta x \rho, \quad (18)$$

where ρ is the number density of interfacial atoms. MD simulations of nuclei r=30 Å, 35 Å, 40 Å were used to obtained the value of ρ, were 0.16020 Å^{-2}, 0.15558 Å^{-2}, 0.15364 Å^{-2} respectively. In FDM simulations, a value of ρ=0.15 Å^{-2} was used for simplicity. Supplementary Fig. S4 shows the interface temperature in MD simulation of nuclei r=40 Å, where 18600 interfacial finite elements, Δx=1 Å, were counted (the black dots), which was consisted with the Gauss distribution (the red line).

It should be noted that, the thermal fluctuation also exist along the circumference. In this case, a finite element, $\Delta x\Delta\theta$, was considered. Here $\Delta\theta$ is a difference element along the circumference. Similarly, as the derivation above, the temperature of this finite element follows the Gauss distribution, and the equation (17), (18) are amended as:

$$\frac{T-T_{x,\theta}}{\sqrt{\frac{2}{3}}T/\sqrt{n}} \propto N(0,1), \quad (19)$$

$$n = r_0 \Delta x \Delta\theta \rho. \quad (20)$$

Supplementary Methods

Extraction of the interfacial profile. In MD simulations, the interfacial profile *r* was extracted to monitor the shrinkage, growth and the instability of the nuclei. To determine the interface profile, we introduced an order parameter ψ_i, proposed by Morries[11]. Firstly, the local order parameter for each atom was calculated via

$$\psi_i = \left| \frac{1}{N_q} \frac{1}{Z} \sum_r \sum_q \exp(iq \cdot r_{ij}) \right|^2 , \tag{21}$$

where N_q is the number of reciprocal vectors of the perfect fcc lattice. In our work, 4 reciprocal vectors along (111) direction were selected. $Z = 12$, denotes the nearest neighbors of each atom *i*, and $r_{ij} = r_j - r_i$ is the vector between two atoms at r_j and r_i. The order parameter is 1 for the perfect solid phase and 0 for liquid phase. To reduce the effects of molecular vibrations and improve the discrimination between the solid and liquid phases, an average order parameter for each atom is used as

$$\bar{\psi} = \frac{1}{Z+1}(\psi_i + \sum_j \psi_j), \tag{22}$$

where *j* runs over all nearest neighbors (Z).

Supplementary Fig. S5 shows the order parameter $\bar{\psi}$ for each atom along the radial direction in a system with coexisting nucleus $r = 30$ Å and liquid at 892.975 K. As seen in the figure, there is $\bar{\psi}>0.4$ in the nucleus region, while the liquid region has the order parameters of $\bar{\psi}<0.2$. Therefore, the atoms whose parameter fall into [0.2, 0.4] were defined as interfacial atoms[11]. In situations of the shrinkage and growth, the interface moved simultaneously, the radius $r(t)$ of the nuclei was calculated by averaging the radius of interfacial atoms.

In the research of the instability, the radius varied greatly on the breach of the nuclei. On this

occasion, the interfaces were divided into series finite element, Δx, along the centerline of the nuclei, and the interfacial atoms belong to every finite element were picked out. Then radii $r(x,t)$ were determined as the average of the interfacial atoms' radius. We consider this method to obtain the interfacial profile is rational, the error analysis was provided in Supplemental Discussion.

Profile of the perturbed interface. According to the equipartition theorem[12] and capillary fluctuation method[13], the relation between the interface profile and interfacial stiffness is:

$$\begin{cases} r(x) = \sum_k A(k)\exp(ikx) \\ \langle |A(k)|^2 \rangle = \dfrac{k_B T}{2\pi r l(\gamma + \gamma'')k^2} \end{cases}, \tag{23}$$

where $k=2\pi K/l$ (K=1,2,3…) is the wave number of fluctuation, l is the length along the centerline of the nuclei. The k-th Fourier modes of the capillary fluctuation, $A(k)$, can be expressed as:

$$A(k) = a_k + ib_k, \tag{24}$$

From equation (23), (24), we can get the expression of the initial interface profile of the perturbed interface:

$$r(x) = r_0 + \sum_{K=1}^{n} 2(a_k \cos kx - b_k \sin kx), \tag{25}$$

where a_k, b_k are random numbers satisfying equation (23), (24).

The approximate Gauss solution of the interface morphology. Once the breaches form on the interface, the effect of the thermal fluctuation on the dynamics of the interface can be neglected, the equation (13) can be simplified:

$$\frac{\mathrm{d}r}{\mathrm{d}t} = \mu\Gamma\frac{\mathrm{d}^2 r}{\mathrm{d}x^2} + \mu\Delta T(1 - \frac{r_0}{r}), \tag{26}$$

substitute r by h ($h=r_0-r$), we get:

$$\frac{\partial h}{\partial t} = \mu\Gamma\frac{\partial^2 h}{\partial x^2} + \mu\Delta T \cdot \frac{h/r_0}{1 - h/r_0}, \tag{27}$$

where

$$\frac{h/r_0}{1-h/r_0} \approx \frac{h}{r_0}, \tag{28}$$

therefore：

$$\frac{\partial h}{\partial t} \approx \mu\Gamma\frac{\partial^2 h}{\partial x^2} + \mu\Delta T \cdot \frac{h}{r_0} \tag{29}$$

The Gauss solution of equation (29) is:

$$h(x,t) = \frac{C_0}{\sqrt{4\mu\Delta Tt + C_1}}\exp(\frac{\mu\Delta Tt}{r_0})\exp(-\frac{\Delta T}{\Gamma}\frac{x^2}{4\mu\Delta Tt + C_1}), \tag{30}$$

where C_0 and C_1 are constants only related to the initial interface morphology.

Dynamics of the interface. To clarify the effect of the thermal fluctuation on the interfacial instability, FDM simulations were performed for nuclei r_0=100 Å under four sets of conditions. The parameters used in FDM are shown in Supplementary Table S2.

Supplementary Discussion

The error analysis of the interfacial profile. In the kinetic expression of finite interface, equation (13), the dependence of the radius on θ, angle along circumference was neglected. Considered this factor, the kinetic expression is amended as

$$\frac{\mathrm{d}r(x,\theta,t)}{\mathrm{d}t} = \mu_\theta \Gamma_\theta \frac{\mathrm{d}^2 r}{\mathrm{d}x^2} + \mu_\theta \eta(x,\theta,t) + \mu_\theta \Delta T(1 - \frac{r_{0,\theta}}{r}) , \tag{31}$$

the subscripts in equation (31) reflect the anisotropy of the interface, such as interfacial energy and kinetic coefficient. According to the work from Mendelev[4] and our previous research[14], this anisotropy is so very weak that can be ignored. What most important is the thermal fluctuation $\eta(x,\theta,t)$, which is uncorrelated in space and time and follows the Gauss distribution (see Supplemental Theories).

Because $r(x,\theta,t)$ is determined by $\eta(x,\theta,t)$, it follows to Gauss distribution, too. In simulations, the nuclei with r = 28 Å, 30 Å**,** 32 Å were relaxed at 892.975 K for 150 ps, the interfacial atoms were picked out for statistics. As showed in Supplementary Fig. S6, the radii tend to be Gauss distribution. From statistics, the 95% confidence interval of r vs. t was calculated, as shows in Supplementary Fig. S7. For the nucleus r = 30 Å, most radii fell into [25 Å, 35 Å], appeared stable in 150 ps; for the nucleus r = 28 Å, who shrank to 20 Å in 150ps, the error of the radii away from the average was no more than 5 Å; the same situation for the nucleus r = 32 Å, who shrank to about 40 Å in 150ps, most interfacial atoms weren't far from their average. Therefore, the method to obtain the radius r is reasonable.

In the research of the instability, the nuclei broke into two parts, gradually. The radius r cannot treated as uniform along the centerline, therefore, expressed as $r(x,t)$. Then the thermal fluctuation along circumference led to deviation of r vs. θ. However, the distribution of interfacial

atoms along circumference still followed to Gauss distribution. Supplementary Fig. S8 shows the interfaces' evolution of the nuclei r = 40 Å at 901.9 K. Three nuclei with different initial interface profiles were relaxed until to rupture. The error bar of 95 % confidence intervals of $r(x,t)$ were given, the rationality of the method to extract $r(x,t)$ was proved again.

The role of fluctuation in the process of instability. The processes of the instability are shown in Supplementary Fig. S9. From Fig. S9a and S9b, the breach emerged approximately at 5 ns for the straight interface with thermal fluctuation, the position of the rupture appeared randomly. For the perturbed interface in Fig. S9c and S9d, the original concave deviation on the interface played the role of the breach, leading to the rupture of the nuclei in 3.12 ns, no apparent difference was observed in spite of thermal fluctuation was introduced. We thus concluded that the thermal fluctuation was important for the formation of the breach and for the determination of the position of the rupture, but did not play a role after the breach formed.

The rapture time of interface. In previous section, the morphology of the rupture was obtained to be an approximate Gauss solution. The evolution of the breach tip is shown in Supplementary Fig. S10, it kept bigger than 70 Å even at 2.5 ns. After that the nucleus broke into two parts promptly in 3.12 ns. When breaking starts, the velocity of the tip can be written as follows:

$$\frac{\partial h(0,0)}{\partial t} = \frac{\mu \Delta T C_0}{\sqrt{C_1}} \cdot \left(\frac{1}{r_0} - \frac{2}{C_1}\right), \tag{32}$$

one can see the tip moves inwards only for $C_1 > 2r_0$. Since C_1 is much bigger than $4\mu\Delta Tt$, equation (30) can then be simplified by introducing $C_1 \approx 4\mu\Delta Tt + C_1$

$$h(x,t) = \frac{C_0}{\sqrt{C_1}} \exp\left(\frac{\mu \Delta T t}{r_0}\right) \exp\left(-\frac{\Delta T}{\Gamma} \frac{x^2}{C_1}\right) \tag{33}$$

where $r_0 = h(0,t)$, further simplification gives:

$$\ln r_0 = \ln \frac{C_0}{\sqrt{C_1}} + \frac{\mu \Delta T t}{r_0}, \tag{34}$$

using equation (5), one can get:

$$\ln r_0 = \ln \frac{C_0}{\sqrt{C_1}} + \frac{\mu \gamma T_{\mathrm{m}}}{L_{\mathrm{V}}} \cdot \frac{t}{r_0^2}, \tag{35}$$

$C_0/\sqrt{C_1}$ is the initial depth of the breaches on the perturbed interfaces, which is only related to the interfacial energy but not to the radius of the nuclei. The depth is around 4 Å for nuclei of r=10 Å to r=10 μm, see Supplementary Fig. S11. In this case, the term $C_0/\sqrt{C_1}$ in equation (35) can be treated as a constant.

With all the material's parameters were brought in, equation (35) can be obtained as:

$$\ln r_0 = 1.42346 + 0.00915 \cdot \frac{t}{r_0^2}, \tag{36}$$

Supplementary Table 3 shows the rupture time of nuclei in FDM simulations. It can be fitted using the relation of $\ln r_0 \propto t/r_0^2$ (see Supplementary Fig. S12):

$$\ln r_0 = 1.78188 + 0.00846 \cdot \frac{t}{r_0^2}, \tag{37}$$

The result in the fitting is very close to the estimation of equation (36), which can be used to predict the rupture time of nuclei with different radius.

Supplementary References

1. Porter, D. A. & Easterling, K. E. *Phase transformations in Metals and Alloys*, 2nd ed. (Chapman & Hall, London, 1992).

2. Bai, X. M. & Li, M. Calculation of solid-liquid interfacial free energy: A classical nucleation theory based approach. *J. Chem. Phys.* **124**, 124707 (2006).

3. Mendelev, M. I., Kramer, M. J., Becker, C. A. & Asta, M. Analysis of semi-empirical interatomic potentials appropriate for simulation of crystalline and liquid Al and Cu. *Phil. Mag.* **88**, 1723 (2008).

4. Mendelev, M. I., Rahman, M. J., Hoyt, J. J. & Asta, M. Molecular-dynamics study of solid-liquid interface miagration in fcc metals. *Modelling Simul. Mater. Sci. Eng.* **18**, 074002 (2010).

5. Wilson, H.A. On the velocity of solidification and viscosity of super-cooled liquids. *Phil. Mag.* **50**, 238 (1900).

6. Frenkel, J. Note on a relation between the speed of crystallization and viscosity. *Physik Z. der Sowjet Union* **1**, 498 (1932).

7. Jackson, K. A. & B. Chalmer. Kinetics of solidification. *Can. J. Phys.* **34**, 473 (1956).

8. Jackson, K. A. Computer modeling of atomic scale crystal growth processes. *J. Cryst. Growth* **198,** 1 (1999).

9. Jackson, K. A. The Interface Kinetics of Crystal Growth Processes. *Interf. Sci.* **10**, 159 (2002).

10. Hoyt, J. J., Trautt, Z. T. & Upmanyu, M. Fluctuations in molecular dynamics simulations. *Math. Comput. Simul.* **80**, 1382, (2010).

11. Morris, J. R. Complete mapping of the anisotropic free energy of the crystal-melt interface in

Al. *Phys. Rev. B* **66,** 144104 (2002).

12. Karma, A. Fluctuations in solidification. *Phys. Rev. E* **48**, 3441 (1993).

13. Hoyt, J. J., Asta, M. & Karma, A. Method for Computing the Anisotropy of the Solid-Liquid Interfacial Free Energy. *Phys. Rev. Lett.* **86**, 5530 (2001).

14. Wu, L. et al. Anisotropic crystal-melt interfacial energy and stiffness of aluminum. *J. Mater. Res*. **30**, 1827 (2015).
